# Real-Time Detection of Circulating Tumor Cells in Bloodstream Using Plasmonic Fiber Sensors

**DOI:** 10.3390/bios12110968

**Published:** 2022-11-03

**Authors:** Shaodi Zhu, Zhenming Xie, Yuzhi Chen, Shiyue Liu, Yiu-Wa Kwan, Shuwen Zeng, Wu Yuan, Ho-Pui Ho

**Affiliations:** 1 Department of Biomedical Engineering, The Chinese University of Hong Kong, Shatin, N.T., Hong Kong 999077, China; 2 Light, Nanomaterials & Nanotechnologies (L2n), CNRS-EMR 7004, University of Technology of Troyes, 10000 Troyes, France; 3 College of Physics and Optoelectronic Engineering, Shenzhen University, Shenzhen 518060, China; 4 School of Biomedical Sciences, The Chinese University of Hong Kong, Hong Kong 999077, China

**Keywords:** circulating tumor cells, fiber optics, surface plasmon resonance, blood, enrichment

## Abstract

Circulating tumor cells (CTCs) are single cancer cells or cancer cell clusters that are present in the circulatory system. Assessing CTC levels in patients can aid in the early detection of cancer metastasis and is essential for the purposes of accurate cancer prognosis. However, current in vitro blood tests are limited by the insufficient blood samples and low concentration levels of CTCs, which presents a major challenge for practical biosensing devices. In this work, we propose the first surface plasmon resonance (SPR) fiber probe to work intravenously, which offers a real-time detection of CTCs in bloodstreams. By exposing the protein-functionalized fiber probe to circulating blood, a continuous capture of CTCs ensures a constant increase in enrichment and hence greatly enhances enumeration accuracy. The performance of our plasmonic fiber probe was demonstrated to specifically detect Michigan Cancer Foundation-7 (MCF-7) breast cancer cells in flowing whole mouse blood. Further, a detection limit of ~1.4 cells per microliter was achieved by using an epithelial cell adhesion molecule (EpCAM) antibody-based receptor layer and a 15 min enrichment period. This pilot study validates real-time CTC detection directly in the bloodstream by using plasmonic fiber probes, which exhibit promising clinical potential for in vivo diagnostic tests involving low concentration biomarkers in circulating blood.

## 1. Introduction

Cancer is one of the leading causes of premature death worldwide. Around 19.3 million new cancer cases and 10.0 million cancer deaths were reported in 2020 [1,2,3,4]. The survivability of a patient is highly related to the stages of cancer. The vast majority of mortality cases are due to the disease metastasizing and multiple new tumors forming away from the initial ones [5,6,7]. Circulating tumor cells (CTCs) are currently known as the key factor for such metastatic cancer. CTCs’ shedding from the primary tumor as single cells, or cell clusters, in the circulatory system allow the cancer to disseminate via the bloodstream [8,9,10]. The number of CTCs presenting in blood has been reported as correlating with cancer progression; further, it is believed that a more advanced cancer would result in higher CTC concentration [10,11]. As CTCs are also found within the early stages of cancer [8,11,12], assessing the CTC level in circulation is, therefore, of great significance for the early detection of cancer and the associated metastasis.

In vitro cell isolation is the first demonstrated approach for diagnostic CTC detection. Vona et al. have proposed a CTC-isolating assay based on the size difference between CTCs and blood cells [13]. Moreover, the immune isolation approach is also widely employed as an alternative to the size-based strategy [14,15]. One example of the immune isolation approach is found in CELLSEARCH (Menarini-Silicon Biosystems, USA), which is a CTC isolating kit approved by the United States Food and Drug Administration (FDA) [16]. This kit utilizes antibody-functionalized magnetic beads in order to selectively separate CTCs overexpressing epithelial cell adhesion molecules (EpCAM). However, the blood sample volume allowed in a single collection from patients is normally in the order of a few milliliters [13,14,15]. Considering the ultra-low concentration level of CTCs in blood, such a small sample volume contains only a limited total number of tumor cells for in vitro CTC assays, leading to a high possibility of false negative results [17,18,19].

To overcome the challenges of in vitro CTC isolation and detection, many researchers have explored the feasibility of evaluating CTC levels after an in vivo enrichment period. This method allows the in situ capturing and accumulation of CTCs in the circulatory system in order to collect more available CTCs for in vitro testing. For example, a venous enrichment at the upper limb of cancer patients can collect tens of cells per second if taking into consideration the high volumetric flow rate in cephalic and basilic veins [20,21]. Such an increased cell number can, in turn, improve the statistical confidence in evaluating the CTC level, leading to a more accurate diagnosis of cancer [19]. One of the representative works considered is an intravascular CTC-isolating device developed by Saucedo-Zeni et al., which is essentially an EpCAM antibody-functionalized guidewire of 0.5 mm in diameter [18]. This intravascular device was demonstrated to help the in vitro CTC detection on breast and non-small cell lung cancer patients after a 30 min venous enrichment procedure. Similarly, Zhang et al. have investigated in vivo CTC enrichment with a functionalized vein indwelling needle and demonstrated its utilities on rabbit and mouse tumor models [22]. It is noteworthy to mention that the abovementioned methods all require post-processing steps such as elution, staining, and counting [18,22,23,24]. These extra procedures may hinder the rapid detection of CTCs. Therefore, a real-time sensing method capable of performing in vivo enrichment is highly desirable in order to achieve the direct detection of CTCs intravenously.

Surface plasmon resonance (SPR) is an appealing method for real-time and label-free biosensor applications [25,26,27]. Recently, there have been some works reported to have used plasmonic sensors for rapid in vitro CTC detection. For example, Mendoza et al. have proposed an SPR microarray for the purposes of analyzing breast-cancer-related proteins and cells in blood. They demonstrated the detection of the membrane markers of breast cancer cells in the suspension of 1 × 10^3^ cells/μL in 30 min [28]. Jia et al. have reported an SPR cytosensor, which is functionalized with a Mucin short variant S1 (MUC-1) aptamer, thereby targeting the Michigan Cancer Foundation-7 (MCF-7) breast cancer cells that are expressing human mucin. They also reported the detection of MCF-7 cells in the suspension of 5 cells/μL after an additional 1 h incubation of NiO-nanoparticles via a SPR signal amplifier [29]. Chen et al. have also reported the detection of MCF-7 cells with an oriented functionalization of peptide on a calixarene monolayer and demonstrated a detection limit of 0.5 cells/μL in 30 min [30].

In this work, we have developed a minimally invasive plasmonic fiber probe, measuring at 125 µm in diameter and intrinsically allowing the intravascular operation through the inner channel of an intravenous (IV) cannula. This plasmonic sensor combines two key functions, including: (i) In vivo enrichment for the purposes of continuously collecting CTCs in circulating blood and (ii) the efficient transmission of light to and from the sensing region into one fiber probe in order to achieve real-time CTC detection in blood vessels. The proposed fiber probe used a hetero-core configuration consisting of a multi-mode fiber (MMF) for the light transmission function, and a gold-coated single-mode fiber (SMF) for the CTC sensing function. A reflective gold layer was coated on the end of the fiber probe in order to allow both light source and SPR interrogation at the same side. The fiber probe was functionalized with an EpCAM antibody for the enrichment and specific detection of MCF-7 breast cancer cells in blood. A homemade blood circulation system was developed in order to mimic the circulating blood flow and to evaluate the performance of the plasmonic fiber probe. In the calibration experiments, the sensors’ sensitivity was measured, via using NaCl solutions, to be 1933.4 nm/RIU. Using a phosphate-buffered saline (PBS) containing MCF-7 cells, the lowest detectable cell concentration was demonstrated to be ~1.4 cells/μL following a 15 min enrichment period. Using heparinized whole mouse blood, we further demonstrated the detection of CTCs in the bloodstream with a concentration of 10 MCF-7 cells/μL within 15 min. The estimated lowest detectable cell concentration in the bloodstream was in consistency with the experiments in the PBS (1.4 cells/μL). These results have, therefore, confirmed the potential of our intravenous plasmonic fiber probe for in vivo enrichment and real-time detection of CTCs in circulating blood.

## 2. Materials and Methods

### 2.1. Chemical Reagents

11-Mercaptoundecanoic Acid (11-MUA) and ethanolamine hydrochloride, polymeric formaldehyde (PFA), and 4′,6-diamidino-2-phenylindole (DAPI) were purchased from Sigma-Aldrich (Hong Kong, China). Dulbecco’s modified Eagle medium (DMEM, 1×), phosphate-buffered saline (PBS, 1×), Trypsin-Ethylenediaminetetraacetic acid solution (Trypsin, 0.25%, phenol red), 1-ethyl-3-(3-dimethylaminopropyl) carbodiimide hydrochloride (EDC), N-hydroxysulfo succinimide (Sulfo-NHS), and the CD326 Monoclonal Antibody (Anti-EpCAM, Clone 1B7) were purchased from Thermo Fisher (Hong Kong, China). The MCF-7 cell line (no. HTB-22) was acquired from ATCC (Manassas, VA, USA). Heparinized mouse (ICR) blood was acquired from the laboratory animal service center in CUHK (Hong Kong, China).

### 2.2. Sensor Surface Functionalization

Step 1: Deposition of 11-MUA monolayer. The sensor was incubated in 10 mM 11-MUA; then, dissolved in ethanol for at least 12 h in order to deposit a carboxylic self-assembling monolayer (SAM). The sensor surface was then rinsed with ethanol and deionized water in order to remove weakly bound molecules.

Step 2: Activation of carboxyl groups. The carboxyl groups on the sensor surface were activated with a 1:1 mixture of 400 mM EDC and 100 mM Sulfo-NHS for 7 min. This step transforms carboxyl groups into amine-reactive NHS esters. Note that EDC and sulfo-NHS should be mixed and used as soon as possible after thawing in order to avoid hydrolysis. The activated senor surface was then rinsed with deionized water in order to remove the extra mixture.

Step 3: Immobilization of the antibody. The activated sensor surface was incubated in a PBS solution containing 25 μg/mL Anti-EpCAM for 2 h. The primary amine groups on the antibody can react with the NHS esters in order to form amide bonds [31]. The antibody-immobilized sensor surface was rinsed with PBS first, and then deionized water second.

Step 4: Blockage of remaining NHS esters. The sensor surface was blocked by 1 M ethanolamine hydrochloride (pH adjusted to 8.5 with HCl) for 5 min to avoid the cross-linking with unspecific proteins during the measurement. The ethanolamine molecules in the ethanolamine hydrochloride solution were bound to the remaining NHS esters though the amine groups [31]. The blocked sensor surface was rinsed with deionized water in order to remove the unbound ethanolamine.

### 2.3. Preparation of CTC Samples

The EpCAM positive cell line MCF-7 was cultured in DMEM in a 95% humidified chamber with a CO_2_ of 5% and at 37 °C for at least 48 h. The cells were harvested using a 5 min Trypsin incubation procedure and gathered in a 15 mL Falcon tube. After centrifugation at 200 g for 5 min, the cells are resuspended in PBS in order to make 2 or 4 mL raw samples. The concentration of raw cell suspension in PBS was calibrated by a hemocytometer (Z359629, Sigma-Aldrich), denoting as C_1_ cells/μL. We added ($C_{1}/500-1)$ mL PBS into 1 mL raw cell suspension in order to obtain the calibrated sample with a concentration of 500 cells/μL for further dilution. 

The diluted MCF-7 cell sample with a concentration of 50 cells/μL in PBS is obtained by adding a 150 μL calibrated sample with a concentration of 500 cells/μL into a 1350 μL PBS. The diluted sample, with a concentration of 10 cells/μL, in the PBS is obtained by adding a 30 μL calibrated sample with a concentration of 500 cells/μL into a 1470 μL PBS. The diluted sample with a concentration of 1 cell/μL in a PBS is obtained by adding a 150 μL sample with a concentration of 10 cells/μL into a 1350 μL PBS.

The diluted MCF-7 cell sample with a concentration of 10 cells/μL in whole mouse blood is obtained by adding a 30 μL calibrated sample with a concentration of 500 cells/μL into 1470 μL blood. Additionally, we added a 30 μL PBS into 1470 μL blood in order to obtain the control sample without MCF-7 cells.

### 2.4. CTC Staining

The CTCs attaching onto the sensor probe are fixed by incubating with a 5% polymeric formaldehyde (PFA) solution for 10 min. After the rinse with the PBS, the fixed cells are stained with a 1 μg/mL DAPI for 5 min. The stained cells are then rinsed with a PBS again. The sensing region of the sensor probe was snapped off with tweezers after DAPI staining and placed in a Petri dish filled with a PBS for the purposes of imaging. The DAPI-stained CTCs can exhibit blue fluorescence, which shows their position on the probe with a 350 nm UV excitation.

### 2.5. Plasmonic Fiber Probe and SPR Interrogation Setup

**Figure 1:**
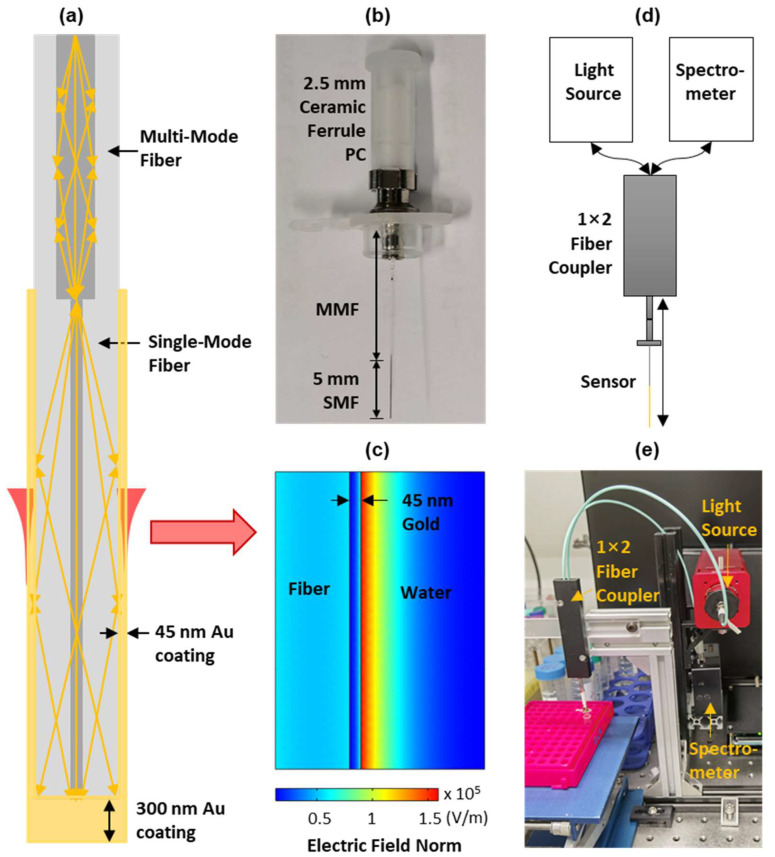
(**a**) Schematic of the plasmonic fiber probe; (**b**) photograph of the fiber probe of a 5 mm long sensing region, coated with a gold thin layer (i.e., the double-headed black arrow); (**c**) simulated electrical field distribution adjacent to the sensor surface of the fiber probe using an excitation wavelength of 600 nm; and schematic (**d**) and photograph (**e**) of the SPR interrogation setup consisting of a fiber probe, a broadband light source, a spectrometer, and a 1 × 2 fiber coupler.

As shown in Figure 1a,b, the fiber probe was fabricated by splicing a 5 mm long SMF to the end of an MMF. Both fibers have a cladding diameter of 125 μm, while the core sizes of MMF and SMF are 62.5 μm and 9 μm, respectively. Due to the core size mismatching, the light guided into the MMF core will leak onto the cladding of the SMF to excite the SPR in the gold-coated thin layer. In our fiber probe, a 45 nm thick gold film was first deposited on the SMF surface using a magnetron sputtering fiber-coating device (JGP450A, SKY Technology Development, Shenyang, China) in order to achieve the balance between sensitivity and signal-to-noise ratio that was previously reported [32]. Then, the gold film on the fiber end was further thickened to 300 nm, which enabled the effective reflection of the sensing light. This reflective probe design allows the placement of a light source and spectrometer at the same side of the probe. By using this method, the probe can pass through the inner channel of a protective tube (IV cannula) and achieve a minimal-invasive CTC detection in vivo.

The sensing mechanism of the plasmonic fiber probe is well established. The light in the fiber sensing region propagates through the total internal reflection will be coupled to the surface plasmon in the gold thin layer via the evanescent field. When the wavevector of light matches that of the surface plasmon, the resonance between them will happen and result in an enhanced electrical field (see Figure 1c), manifested as a strong absorption dip in the reflected SPR spectrum, i.e., an SPR dip. When biomolecules, or cells, of a different refractive index to background medium attach to the sensor surface, there are changes to the resonance condition between the light and surface plasmon, which causes the shift of the SPR dip [26,33]. This sensing mechanism is used in our plasmonic fiber sensor in order to detect CTCs in circulating blood in real-time by continuously interrogating the reflected spectra.

The schematic and a photograph of our SPR interrogation system are shown in Figure 1d,e. This system includes a broadband light source (SLS201L, Thorlabs, Newton, NJ, USA), a spectrometer (USB 4000, Ocean Optics, Dunedin, FL, USA), and a 50:50 multi-mode coupler (bandwidth: 600 ± 50 nm, Optics Forest, Huizhou, China). In this system, the light will be coupled into the fiber probe through the fiber coupler in order to excite the surface plasmon polariton (SPP) in the gold sensing region. The sensing light will then be reflected backwards by a thickened gold layer on the fiber end and will re-excite the SPP in the sensing region before it is measured by the spectrometer.

### 2.6. Blood Circulation Mimicking System

**Figure 2:**
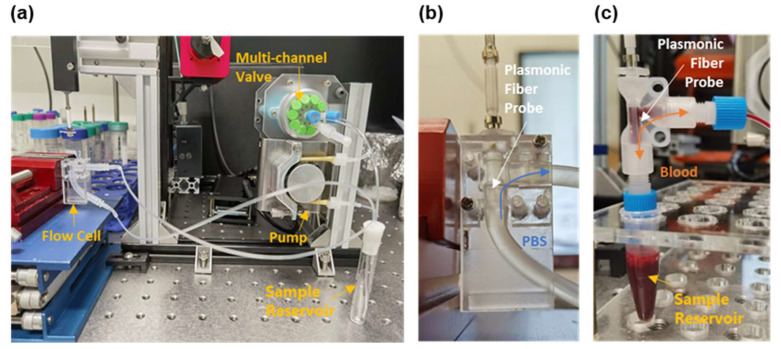
(**a**) Photograph of the homemade blood circulation mimicking system, which consists of a peristaltic pump, a multi-channel valve, a sample reservoir, and a flow cell that is 2.4 mm in diameter. Zoom-in view of the flow cell in (**a**) for calibration experiments using PBS (**b**) and the targeting experiments using whole mouse blood (**c**). In (**b**,**c**), the white arrows indicate the plasmonic fiber probe in the flow cell, the blue arrow denotes the flow direction of PBS, and the orange arrow represents the reciprocating direction of the blood flow. An additional sample reservoir is placed below the flow cell designed for blood (**c**) to buffer the reciprocating flow.

As shown in Figure 2a, a homemade blood circulation system has been developed to mimic the blood flow of a controllable volumetric flowrate. This system consists of a homemade peristaltic pump, a multi-channel valve (SV-03, Runze Fluid, Nanjing, China), a sample reservoir, and a flow cell hosting the fiber probe. The inner diameter of the flow cell is 2.4 mm (see Figure 2b), close to that of the cephalic vein commonly used to place intravascular catheters [34]. The analyte volume surrounding the sensing region is about 22.6 μL. The volumetric flowrate was limited to 3.0 μL/s in order to avoid the rupture of cells by shear stress [35,36]. In the calibration experiments that used PBS solutions, the cell samples were continuously circulated through the flow cell in one flow direction (see Figure 2b). In the targeting experiments that used whole mouse blood, the blood samples were reciprocated in the flow cell in order to fully utilize the limited blood volume (1.5 mL) that was collected from mice (see Figure 2c).

## 3. Results and Discussion

### 3.1. Evaluation of the Sensitivity of Plasmonic Fiber Probe

**Figure 3:**
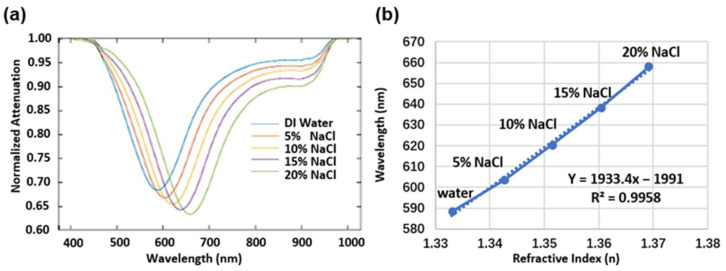
(**a**) SPR spectra obtained for the purposes of measuring NaCl solutions with different concentrations from 0 to 20% (*w*/*w*%) in deionized (DI) water. (**b**) Wavelength values (solid line) of the SPR dip changing with the refractive index of the NaCl solutions [37]. The dash line is the linear fitting of the wavelength values.

In our studies, the normalized SPR spectra were achieved by dividing the reflected sensing spectra to a pre-acquired reference spectrum of the light source (see Figure S1) and the change in the SPR dip wavelength was monitored and recorded in real time [26]. Figure 3a illustrates the change in the SPR spectra with NaCl solutions of different concentrations, ranging from 0% to 20% in weight ratio in deionized water, which indicate the probe’s sensing response to solutions of variant refractive indices [37]. The SPR dip shifts to a longer wavelength accompanying a stronger optical attenuation with the increased refractive index (see Figure 3b and Figure S2). In general, the sensitivity (S) of the SPR sensor is given by the ratio of shifted wavelength (dλ) to refractive index change (dn), i.e., S=dλ/dn. Therefore, as shown in Figure 3b, a linear fitting was performed in order to characterize the sensitivity of the plasmonic fiber probe to be 1933.4 nm/RIU. As the resolution of the used spectrometer is 0.2 nm, the limit of detection (LOD) of the fiber probe is about 1 × 10^−4^ RIU. 

### 3.2. Functionalization of the Plasmonic Fiber Probe

**Figure 4:**
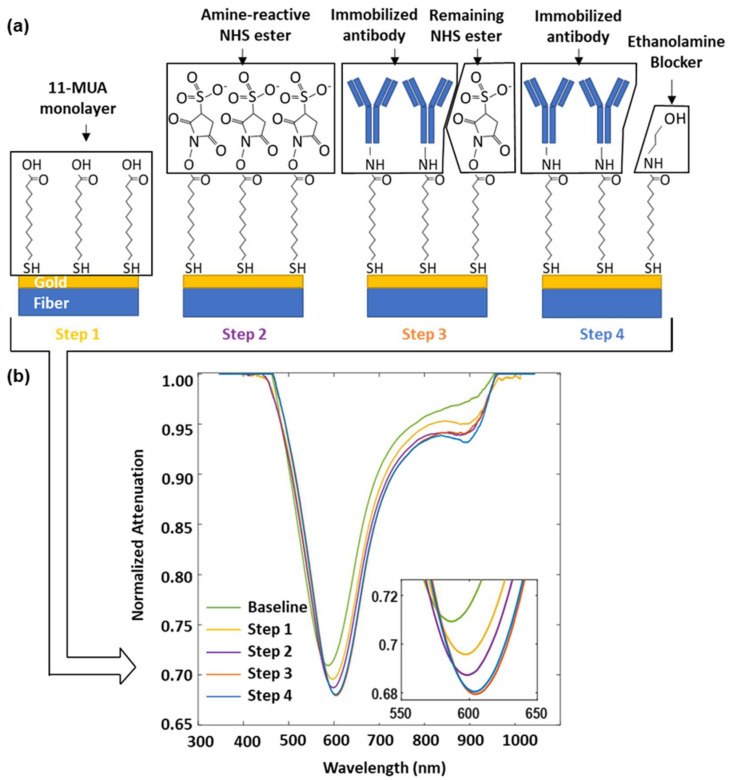
(**a**) Schematic of the surface functionalization of the plasmonic fiber probe for targeted MCF-7 cell sensing. Step 1: Form a self-assembling monolayer of 11-MUA on gold. Step 2: Activate the carboxyl groups on 11-MUA through the EDC/NHS reaction. Step 3: Immobilize the EpCAM antibody for the purposes of cell capture. Step 4: Block remaining active NHS esters. (**b**) SPR spectra recorded at the end of each functionalization step, inset shows the zoomed in view of the SPR dips.

The enrichment and detection of CTCs is based on the affinity binding between the EpCAM that is overexpressed on the cancer cell membrane (MCF-7), as well as the EpCAM antibody (anti-EpCAM) immobilized on the sensor surface of the plasmonic fiber probe. A detailed surface fictionization protocol is discussed in the Materials and Methods section. In essence, as shown in Figure 4a, the antibody immobilization procedure begins with forming a self-assembled alkanethiol layer with a carboxyl group decoration (11-MUA) on the gold surface. Then, the EpCAM antibody is grafted to this carboxylic end through EDC and a sulfo-NHS mediated crosslinking reaction. After blocking the remaining active NHS esters with ethanolamine, the sensor surface is ready to offer a specific affinity targeting of EpCAM-associated MCF-7 cells. 

Figure 4b shows the change in SPR dips during the surface functionalization process. The molecule attachment on the sensor surface resulted in a red-shifted SPR dip accompanied with an attenuated optical power, which was observed in the steps of 1, 2, and 3 for the deposition of 11-MUA, the decoration of sulfo-NHS ester, and the immobilization of the antibody, respectively. It was also found that the ethanolamine blocking in step 4 leads to a slightly inversed change, which may be caused by the hydrolysis of an unreacted sulfo-NHS ester remaining on the sensor surface. The wavelength of the SPR dip shifts from 586.7 nm to 604.1 nm during the functionalization process, leading to a change of 17.4 nm in total.

### 3.3. CTC Enrichment and Sensing in PBS Flow

**Figure 5:**
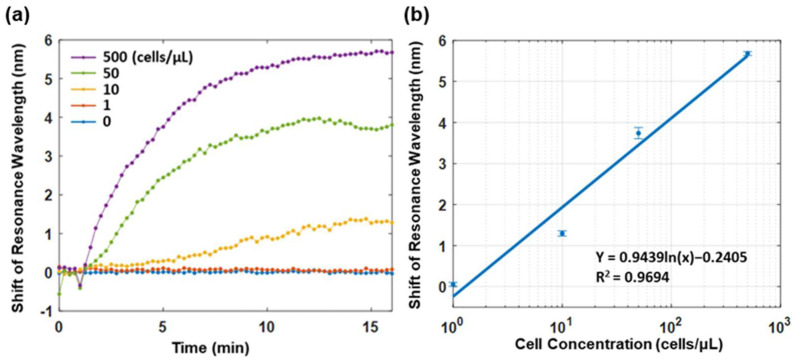
(**a**) SPR sensorgrams monitoring the relative shift of the resonance wavelength of the functionalized plasmonic fiber probe in PBS flow of different MCF-7 cell concentrations in real time. (**b**) The logarithm fitting of the wavelength shifts of SPR dip versus variant MCF-7 cell concentrations. The data points denote the relative shift of resonance wavelength recorded at the end of a 15 min enrichment in each sample. The error bar shows the standard deviation of data points in one minute before the end of enrichment.

Figure 5a illustrates the sensorgrams of the functionalized plasmonic fiber probe in the continuous PBS flow containing MCF-7 cells of different concentrations from 0 to 500 cells/μL. The sensorgram was acquired at a rate of 20 data points per second and plotted the averaged data by every 15 s. It was found that the sensorgram reached the saturation after a wavelength shift of 5.6 nm in 15 min when testing the PBS flow with a concentration of 500 MCF-7 cells/μL; this was while the wavelength shift at 15 min were 3.8 nm and 1.3 nm for the PBS flows with a concentration of 50 and 10 MCF-7 cells/μL, respectively. It is also noted that there is only a marginal difference of about 0.1 nm in terms of the shift of resonance wavelength between the sensorgrams of the PBS flow of 1 MCF-7 cell/μL and PBS baseline. 

To evaluate the LOD of the functionalized plasmonic fiber probe, a logarithm fitting was performed on the relative shift of resonance wavelength recorded after a 15 min enrichment period versus PBS solutions of different MCF-7 cell concentrations. By using the fitted logarithm equation and considering the spectrometer resolution of 0.2 nm, the LOD of the fiber probe is about 1.4 MCF-7 cells/μL in PBS. Considering that the used volumetric flow rate in the flow cell was 3 μL/sec. The lowest detectable cell flow rate of the fiber probe is approximately 4.2 cells/sec, which is in the same order of the cell flow rate in the cephalic vein of cancer patients [11,20].

To investigate the specificity of the plasmonic fiber probe, the sensing responses of fiber probes functionalized with and without anti-EpCAM were studied using a PBS flow with a concentration of 500 MCF-7 cells/μL. In a 15 min sensing period, the relative shift of the resonance wavelength of the fiber probe functionalized with an antibody is found to be almost three times of that measured with the fiber probe without an antibody (see Figure S3a). To further demonstrate the unspecific binding resistance of an ethanolamine-blocked 11-MUA layer on the plasmonic fiber probe, the sensorgram of a fiber probe functionalized with a blocking layer was compared with that of a fiber probe without a blocking layer (i.e., a bare gold sensor surface). As no anti-EpCAMs were grafted on these two fiber probes, the detected resonance wavelength shifts in both sensorgrams were only contributed by the unspecific cell bindings. As shown in Figure S3b, the blocking layer successfully suppressed the unspecific cell bindings by about 50%, as indicated by the relative shifts of resonance wavelength of the two fiber probes measured at 15 min. 

**Figure 6:**
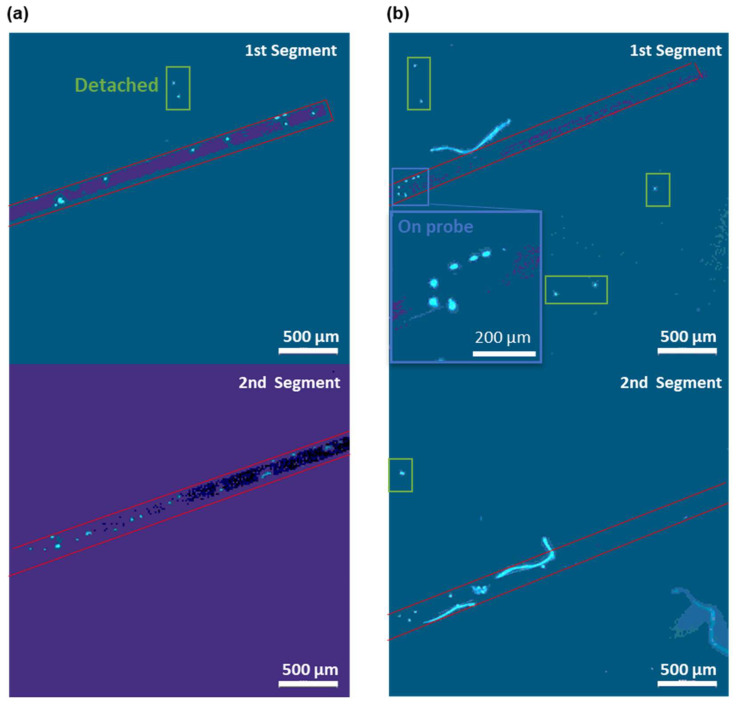
Micrographs of DAPI-stained MCF-7 cells (in bright blue color) on the fiber probes after the 15 min enrichment in PBS flows with a concentration of (**a**) 500 and (**b**) 10 MCF-7 cells/μL. Two 2.5 mm segments of the 5 mm long sensing region of the fiber probe were imaged separately with the 1st segment close to the fiber end and the 2nd segment close to the MMF. The red boxes outline the fiber probes in each micrograph. Insets show the zoomed in view of the cells attached on the probe surface (blue boxes). The detached cells are marked by green boxes.

As shown in Figure 6, after the real-time cell detection, we have taken out the sensing part of the probe via a tweezer in order to image and validate the attached cells. The DAPI staining led to a blue fluorescence of DNA in nucleus. Hence, the cells attached to the probes can be visualized as bright blue spots via fluorescence microscopy. By counting the number of bright spots on the fiber surface, we validated that at least 29 cells were attached to the probe in Figure 6a, after a 15 min enrichment PBS flow with an MCF-cell concentration of 500 cells/μL. In Figure 6b, the attached cell number on the fiber probe is decreased to the number of 12 due to the lower MCF-7 cell concentration of 10 cells/μL in the enriching PBS flow. We have only counted the cell number on one side of the probe due to the fact that the gold film is not transparent under the microscope. Therefore, the total cell number on the probe should be doubled if the specific binding of CTCs is symmetric on both sides of the fiber probe. The total cell number on the probe is 58 for the enrichment in PBS flow with a concentration of 500 cells/μL and 24 for 10 cells/μL. To enable the number of the attached cells to be more precise, the large bright spots of multiple overlapping cells are counted as one cell in Figure 6. Moreover, the bright spot overlapping with the impurity is not counted. Additionally, there were some cells that detached from the fiber probe during the staining procedure (see green boxes in Figure 6). These detached cells were not counted as attached cells and the weak affinity of them may be due to the unspecific attachment on the non-sensing region. In addition, the cells have been fixed with formaldehyde to ensure their attached positions on the fiber probe and in order to remained in the same spot. 

These micrographs of the CTCs attached on the probe surface confirmed that the total cell number on the probe is also positively correlated with the shifts in the resonance wavelength. There were 58 cells attached to the probe and a 5.6 nm shift in the resonance wavelength on the sensorgram for the enrichment in PBS with a concentration of 500 MCF-7 cells/μL. Similarly, for 10 MCF-7 cells/ cells/μL, the total cell number on the fiber probe was 24 and a resonance wavelength shift of 1.3 nm was found. No cell was found on the fiber probe following the sensing in the PBS flow of 1 MCF-7 cell/μL (see Figure S4), which is consistent with the marginal resonance wavelength shift observed in Figure 5a. Thus, in addition to the real-time CTC detection based on the SPR wavelength shift, our SPR fiber probe can also effectively differentiate the patients with cancer by directly determining the CTC existence after enrichment [10,11]. 

### 3.4. CTC Enrichment and Sensing in Circulating Blood

**Figure 7:**
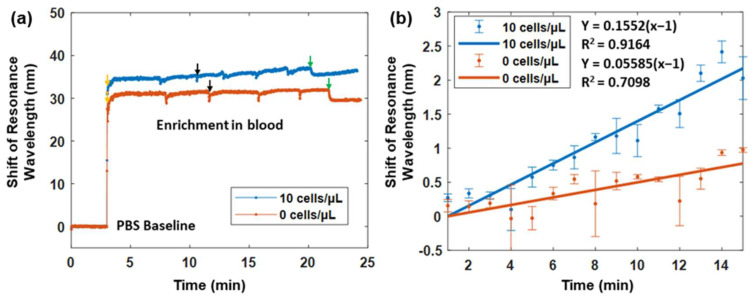
(**a**) SPR sensorgrams of the plasmonic fiber probe in the reciprocating whole mouse blood with a concentration of 10 MCF-7 cells/μL (blue line) versus that in the reciprocating mouse blood only (orange line). Both probes were first immersed in PBS solutions before the introduction of a reciprocating blood flow, as indicated with arrows. Black arrows indicate the change in flow direction by the peristaltic pump, and green arrows denote the halt of pumping. (**b**) The sensorgrams in (**a**) are smoothed through the averaging of each minute during the 15 min enrichment period. The blue and orange points denote the average sensing responses of two fiber probes in whole mouse blood with MCF-7 cells and without MCF-7 cells, respectively. The error bar refers to the standard deviation of the signal when one minute has passed. Linear fittings are performed in order to illustrate the sensing dynamics of two fiber probes in blood samples with MCF-7 cells (blue line) and without MCF-7 cells (orange line).

We further evaluated the performance of the plasmonic fiber probe in the context of circulating whole mouse blood in order to evaluate its clinical potential in complex physiologic matrices. As shown in Figure 7a, when comparing with the PBS baseline, the resonance wavelength of the fiber probes in the circulating blood with a concentration of 0 and 10 MCF-7 cells/μL were shifted to about 29.8 nm and 33.1 nm, respectively. Due to the reciprocated blood flow in the flow cell, transient signal drifts caused by the change in flow direction were observed in Figure 7a. The shift in the resonance wavelength increased during the enrichment of the fiber probe in the blood sample containing CTCs, leading to a total wavelength shift of 2.2 nm after 15 min versus a 0.7 nm wavelength shift in the non-CTCs control study. 

To better visualize the sensing dynamic of the plasmonic fiber probe during the 15 min enrichment, the shift of the resonance wavelength was averaged for every one minute in order to remove the drifts (see Figure 7b). Linear fittings were then performed. The resonance wavelength of the fiber probe was found to change almost three times faster in the circulating blood of CTCs, compared to that in the non-CTCs control. After the 15 min enrichment, the difference of the wavelength shift between the two cases was about 1.5 nm, which is consistent with the 1.3 nm wavelength shift found when testing the PBS flow with a concentration of 10 MCF-7 cells/μL. Considering the spectrometer resolution, the LOD of the fiber probe for CTC detection in circulating blood is also at the similar level of ~1.4 MCF-7 cells/μL.

## 4. Conclusions

In this work, we have demonstrated a novel real-time CTC detection approach, which can directly measure in the bloodstream through a needle-like plasmonic fiber probe. Compared to the conventional SPR sensor-based (on a Kretschmann configuration) approach, this fiber probe is compact and intrinsically compatible for the purposes of intravenous diagnoses. In addition, thanks to the scheme of continuous cell enrichment, the available sample volume is not a limitation in our approach, as it is in the standard in vitro CTC isolating methods [13,14,15,16]. Moreover, when comparing to current intravenous CTC-enriching devices—which are based on the medical wire [18,24] and the indwelling needle [22,23]—our fiber probe is able to provide a real-time detection of CTCs with an optical signal transmission through an optical fiber.

The sensitivity of our plasmonic fiber probe for the purposes of CTC detection is 1933.4 nm/RIU in the refractive index range from 1.33 to 1.37, which is better than most of the reported sensitivities of the hetero-core-based SPR fiber sensors (ranging from 259.85 nm/RIU to 1561 nm/RIU) [38,39,40,41,42,43]. The CTC detection limit in the bloodstream is estimated to be 1.4 cells/μL, which is comparable to the LODs reported in previous works [28,29,30]. It is worthwhile to note that our fiber probe can specifically detect CTCs in the bloodstream and was able to demonstrate a three times stronger and faster change in the plasmonic resonance wavelength in blood samples containing CTCs versus those in blood samples without CTCs. In the long run, we envision that this CTC detection approach, using a plasmonic fiber probe, can be integrated into the cancer treatments that are based on an infusion approach, thereby allowing a tracking of treatment efficacy. Furthermore, compared to the current SPR device, which has a bulky size (~100 cm × 50 cm × 50 cm) and high cost (~500 k dollars for GE Biacore 4000), our plasmonic fiber probe is low profile and low cost, suitable for disposable use in cancer screening and in other points of care testing. 

The current study simply demonstrated the feasibility of the plasmonic fiber probe for the purposes of CTC detection in the bloodstream. It was limited to detect CTCs in blood samples. A systematic study is imperative in order to validate the clinical potential of plasmonic fiber sensors for detecting CTCs in animal models and patients. For future clinical use, the sensitivity of our fiber probe needs to be further improved for an accurate CTC detection of early cancers; this is because the detection of early cancers requires a LOD of CTC concentrations of less than 0.1 cells/μL in the bloodstream [8,11,12]. To improve the sensitivity and LOD, we will optimize the design of our plasmonic fiber probe by using an enhanced plasmonic substrate based on the metallic bilayers of optimized thicknesses [44]. We will also explore the use of monolayers of two-dimensional materials in our fiber probe allowing for the tailoring of the optical absorption in sub-nanometer scale [45,46] in order to achieve a plasmonic sensitivity over 105 nm/RIU. In addition, we will further improve the functionalization protocol by using captured antibodies of controlled orientation on the probe surface in order to enhance their capture efficiency. For example, Sharma et al. has used a reducing agent, TCEP, in order to split the capture antibody in the middle so as to generate a thiol group on the other side of the fragment for binding to the probe surface [47]. Thus, the capture fragment antigen-binding region (Fab region) can be highly oriented. Another surface functionalization solution is to introduce a layer of receptors that selectively bind to the tail of the antibodies, which allows the antibodies to be arranged upward [48,49]. 

## Data Availability

All data needed to evaluate the conclusions in the paper are present in the paper and/or the Supplementary Materials. Additional data related to this paper are available upon request.

## Supplementary Materials

The following supporting information can be downloaded at: https://www.mdpi.com/article/10.3390/bios12110968/s1, Figure S1: The signal extraction process of SPR dip; Figure S2: The shift of SPR dip intensity versus refractive index via the testing of NaCl solutions of different concentrations; Figure S3: (a) Comparison of MCF-7 sensing performances between the sensors functionalized with and without an EpCAM antibody. (b) Comparison of the unspecific binding of MCF-7 cells between the blocked and unblocked sensors. Figure S4: Micrographs of the sensing area after the enrichment in flowing PBS containing 1 cells/μL.
