# Supplementary material for: Real-Time Detection of Circulating Tumor Cells in Bloodstream Using Plasmonic Fiber Sensors
Source: Biosensors (Basel). 2022 Nov 3;12(11):968. doi: 10.3390/bios12110968 (PMC9687831; doi:10.3390/bios12110968)
Supplement: Supplementary file 1 [file biosensors-12-00968-s001.zip › biosensors-1961248-supplementary.pdf]

biosensors

Supplementary Materials

Real-Time Detection of Circulating Tumor Cells in Bloodstream Using Plasmonic Fiber Sensors

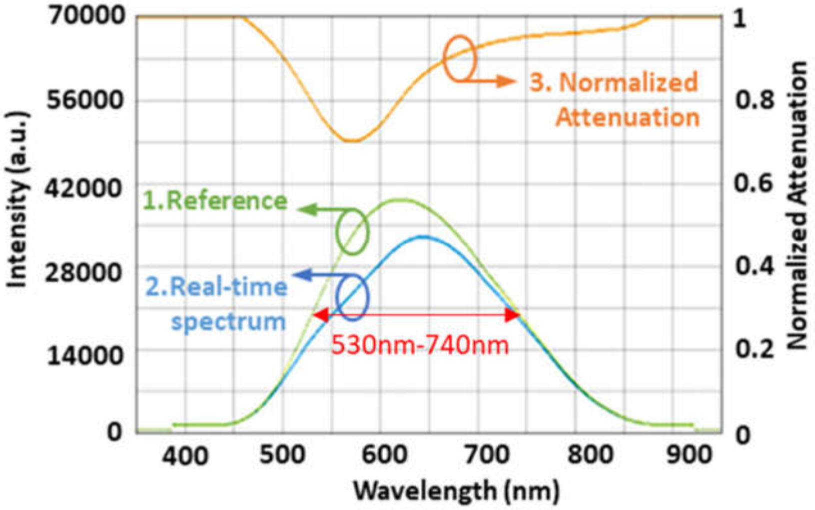

Figure S1. The signal extraction process of SPR spectrum. Step 1: Record a reference spectrum in air (green Line). The bandwidth of reference spectrum is marked by red double arrow. Step 2: Record the real-time sensing spectrum continuously during the enrichment (blue line). Step 3: Calculate the normalized attenuation of the real-time sensing spectrum (Orange line) by dividing with reference spectrum. The orange line is the resulting SPR spectrum.

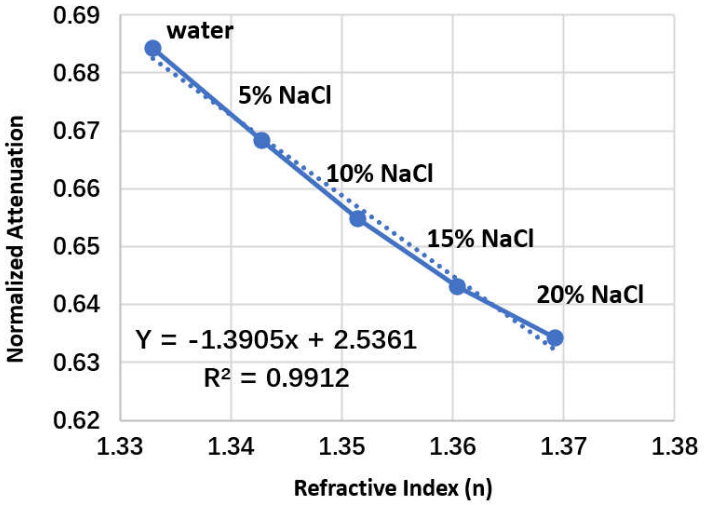

Figure S2. The shift of SPR dip intensity versus refractive index by testing NaCl solutions of different concentration. This indicator was not used for evaluating the sensor performance since it has a lower detection limit of 5x10^{-4}RIU, which is constrained by the power drift of broadband source.

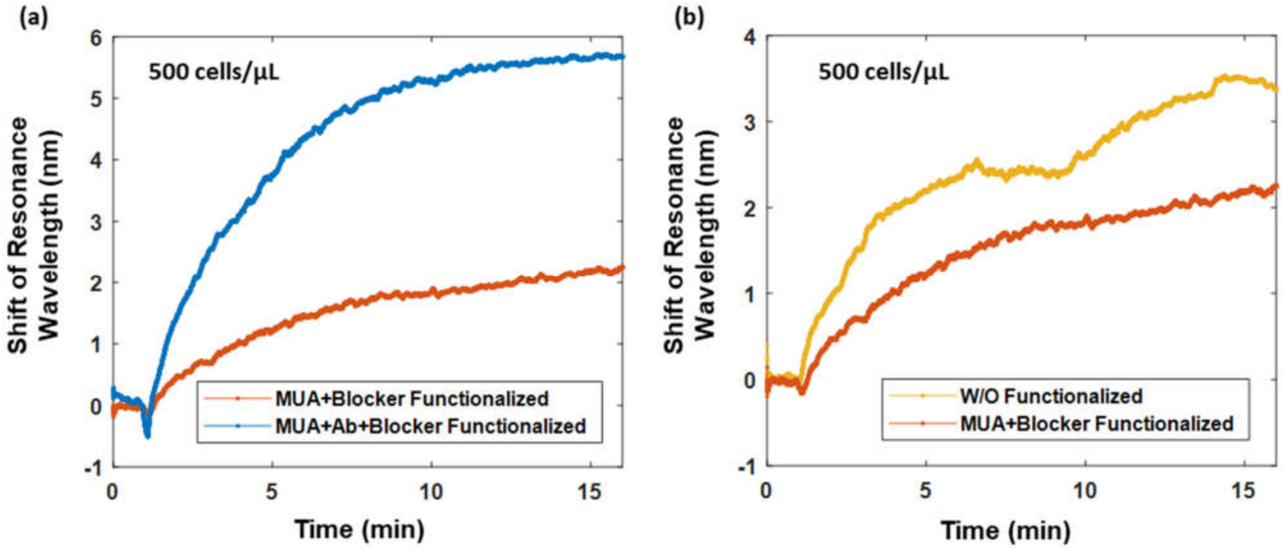

Figure S3. (**a**) Comparison of MCF-7 sensing performance between the sensors functionalized with and without EpCAM antibody. Sensor functionalized with antibody (blue line) has much higher SPR signal shift than non-antibody sensor (orange line). (**b**) Comparison of the unspecific binding of MCF-7 cells between the blocked and unblocked sensors. The sensor functionalized with an ethanolamine blocked 11-MUA layer (orange line) showed reduced signal shift in comparison with an unblocked sensor without any functionalization (yellow line).

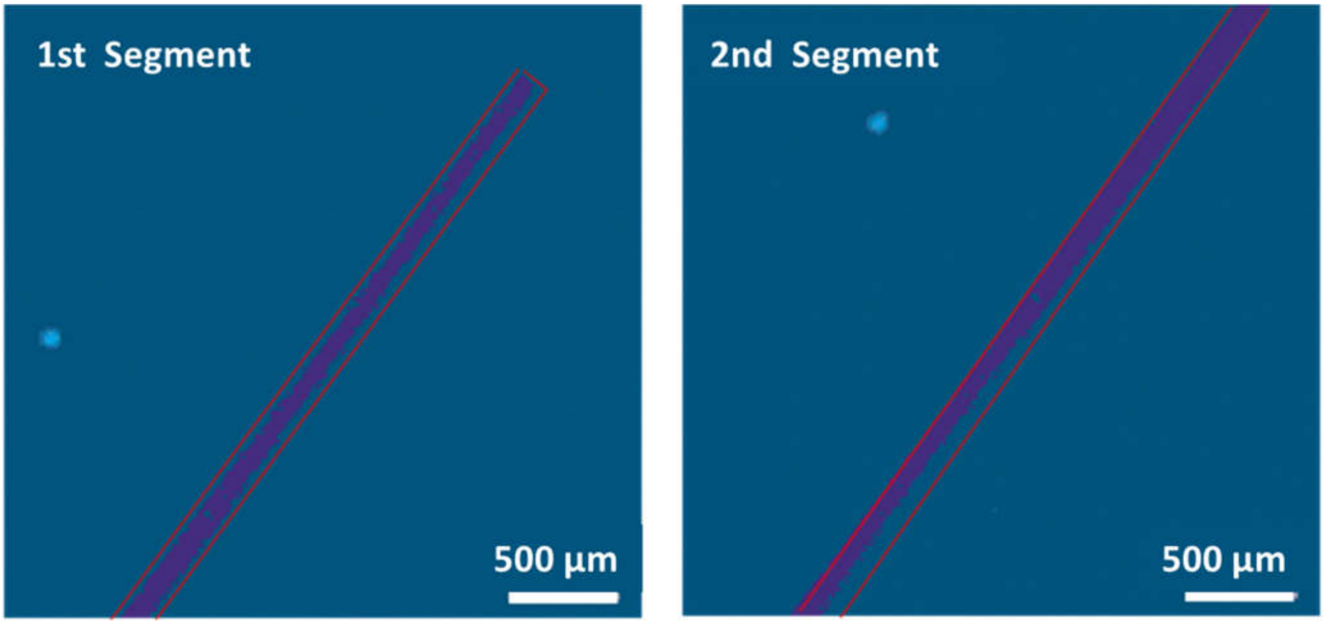

Figure S4. DAPI Staining micrographs of the sensor probe without any observable cell binding after the enrichment test in PBS flow (Figure 5). It is due to the ultralow cell concentration of 1 cell per microliter. The first Segment of micrograph illustrates the sensing region (2.5 mm) close to the fiber end. The second segment (2.5 mm) include the remaining parts of the sensing region. The sensor probes have been outlined by red lines.
